# Supplementary material for: Monozentrische Phase‐IIb‐Studie zur Wirksamkeit von Apremilast beim nummulärem Ekzem
Source: J Dtsch Dermatol Ges. 2025 Aug 11;23(8):959–66. [Article in German] doi: 10.1111/ddg.15786_g (PMC12338416; doi:10.1111/ddg.15786_g)
Supplement: Supplementary file 1 — Supplementary information [file DDG-23-959-s001.docx]

**JDDG-2024-OA-626.R3_Eyerich_GER**

**Query/ Note to the author:**

- Q1: Please disclose any conflicts of interest for all authors.

**Monozentrische Phase-IIb-Studie zur Wirksamkeit von Apremilast bei nummulärem Ekzem**

Alexander Böhner^1^, Peter Seiringer^1^, Viktoria Lang^1^, Danielle Rogner^1^, Christian Oesterlin^2^, Tilo Biedermann^2^, Kilian Eyerich^3,4*^, Felix Lauffer^1*^

^1^Klinik und Poliklinik für Dermatologie und Allergologie, Technische Universität München, München, Deutschland

^2^Dermazentrum, Freiburg, Deutschland

^3^Klinik für Dermatologie und Venerologie, Universitätsklinikum Freiburg, Freiburg, Deutschland

^4^Abteilung für Dermatologie und Venerologie, Department of Medicine Solna, sowie Zentrum für Molekulare Medizin, Karolinska Institutet, Stockholm, Schweden

*Beide Autoren haben gleichermaßen zu dieser Arbeit beigetragen.

**Kurztitel:** Wirkung von Apremilast beim nummulären Ekzem

Korrespondenzanschrift:

Dr. med. Alexander Böhner

Klinik und Poliklinik für Dermatologie und Allergologie

Technische Universität München

Biedersteinerstraße 29

80802 München

E-Mail: alexander.böhner@tum.de

Zusammenfassung

**Hintergrund:** Über die Pathogenese des nummulären Ekzems (NE) ist wenig bekannt und es gibt keine zugelassene zielgerichtete Therapie. Apremilast ist ein Phosphodiesterase-4-Inhibitor.

**Patienten und Methodik:** Eine randomisierte, doppelblinde, placebokontrollierte Phase-IIb-Studie zur Bewertung der Wirkung von Apremilast bei Patienten mit NE. Die Patienten erhielten Apremilast (30 mg zweimal täglich) oder Placebo bis Woche 16, gefolgt von einer offenen Phase, in der alle Patienten bis Woche 32 mit Apremilast behandelt wurden. Der primäre Endpunkt war die Anzahl der Patienten, die in Woche 16 eine Verbesserung des *Physician Global Assessment* (PGA) um mindestens 2 Punkte oder einen absoluten PGA-Wert von 0 oder 1 erreichten. Zu den sekundären Endpunkten gehörten Veränderungen der Hautphysiologie, Dermatopathologie und Lebensqualität.

**Ergebnisse:** 33 Patienten wurden in die Studie aufgenommen, von denen 31 Patienten in die Apremilast (n = 15) oder Placebo (n = 16) Gruppen randomisiert wurden. Der primäre Endpunkt wurde bei 1/15 (6,7 %) in der Apremilast-Gruppe und 4/16 (25,0 %) Patienten in der Placebogruppe erreicht (p = 0,369). In Bezug auf alle sekundären Endpunkte gab es in Woche 16 und Woche 32 keinen Unterschied zwischen Placebo und Apremilast. Das Sicherheitsprofil entsprach dem bekannten Sicherheitsprofil von Apremilast.

**Schlussfolgerung:** Die Hemmung der Phosphodiesterase-4 durch Apremilast zeigte keine positiven Auswirkungen auf die Behandlung von NE.

**SCHLÜSSELWÖRTER**

Nummuläres Ekzem, nummuläre Dermatitis, Apremilast, Phosphodiesterase-4-Inhibitor, klinische Studie, Apreminum

**EINLEITUNG**

Das nummuläre Ekzem (NE) ist eine chronisch-entzündliche Hauterkrankung (inflammatory skin disease, ISD), die durch juckende, nummuläre Ekzeme gekennzeichnet ist. Diese treten am häufigsten an den oberen und unteren Extremitäten auf.^1,2^ Das nummuläre Ekzem wurde 1854 erstmals von Devergie beschrieben.^3^ Synonyme sind diskoides Ekzem, mikrobielles Ekzem und nummuläre Dermatitis. Das nummuläre Ekzem nimmt oft einen chronischen und rezidivierenden Verlauf, ist in der Regel schwer zu behandeln, juckt stark und beeinträchtigt die Lebensqualität erheblich.^4^ Die Diagnose wird klinisch in Korrelation mit histologischen Befunden gestellt. Die Ätiologie des nummulären Ekzems ist bislang nicht eindeutig geklärt; charakteristisch ist jedoch eine Kombination aus gestörter epidermaler Barriere, Hautentzündung und mikrobieller Besiedlung.^4,5^ Klinisch und histologisch zeigt das NE Überschneidungen mit der atopischen Dermatitis (AD), der Kontaktdermatitis sowie mitunter auch mit der Psoriasis.^4^

Die Erkrankung tritt in allen Lebensabschnitten auf. Aus der Literatur geht hervor, dass Frauen etwas häufiger betroffen sind als Männer. Über die Häufigkeit von NE ist wenig bekannt. Eine Studie berichtet über eine Prävalenz von bis zu 9 % bei alkoholkranken Patienten in Brasilien und eine Punktprävalenz von etwa 2 % in der Allgemeinbevölkerung.^4^ Das nummuläre Ekzem tritt häufiger bei Patienten mit atopischen Erkrankungen (AD, allergisches Asthma oder Rhinokonjunktivitis) auf. Weitere Triggerfaktoren sind chronische Kontaktdermatitis, mikrobielle Hautbesiedlung, Behandlung mit systemischen Retinoiden oder Alkoholmissbrauch.^1,2,4–6^ Ein generalisiertes nummuläres Ekzem wurde im Zusammenhang mit einer Interferon-/Ribavirin-Therapie bei Hepatitis C beschrieben.^7^ Es gibt nur wenige therapeutische Optionen für NE, darunter topische und systemische Kortikosteroide, topische Calcineurininhibitoren und Phototherapie.

Über die Pathophysiologie des NE ist wenig bekannt. Aufgrund der Assoziation mit atopischen Erkrankungen, erhöhten Serum-IgE-Werten, Juckreiz und mikrobieller Besiedlung betrachten viele Autoren das NE als eine Variante der AD. Noda et al. berichteten, dass die asiatische Variante der AD häufig ähnliche klinische Merkmale wie das NE aufweist, wie eine scharfe Abgrenzung und eine nummuläre Morphologie. Interessanterweise ist die asiatische AD auf der Ebene der Genregulationen zwischen der Psoriasis und der klassischen AD angesiedelt. Darüber hinaus zeigte die asiatische AD eine stärkere Akanthose und eine höhere Keratinozyten-Proliferation, gemessen durch Ki67-Färbung, als die klassische AD. Während in beiden Fällen eine Hochregulation von Genen nachgewiesen wurde, die mit T-Helferzellen vom Typ 2 (Th2) assoziiert sind, zeigte sich ein signifikanter Anstieg der Expression von *IL17A*, *IL19* und *S100A12* ausschließlich in Hautbiopsien der asiatischen atopischen Dermatitis.^8^ Angesichts der klinischen Ähnlichkeit von NE und asiatischer AD ist es wahrscheinlich, dass NE ebenfalls auf einer gemischten Th2/Th17-Immunregulation beruht. Dieses Konzept wird durch die Tatsache unterstützt, dass NE gut auf Therapien anspricht, die sowohl bei Psoriasis als auch bei AD wirksam sind, wie UV-Therapie und Methotrexat.^9^ Bislang gab es jedoch weder klinische Untersuchungen an größeren NE-Patientenkohorten noch kontrollierte klinische Studien, in denen neue Wirkstoffe beim NE getestet wurden. Um ein besseres Verständnis der dem NE zugrunde liegenden immunologischen Prozesse zu gewinnen, sind Daten aus randomisierten, kontrollierten Studien unerlässlich.

Apremilast ist ein *small molecule*, das selektiv die Aktivität der Phosphodiesterase 4 (PDE4) hemmt – der zentralen Enzymklasse, die für den Abbau von zyklischem Adenosinmonophosphat (cAMP), einem intrazellulären Botenstoff, verantwortlich ist. PDE4 wird in Entzündungs- und Epithelzellen, wie Keratinozyten, exprimiert.^10^ PDE4-Inhibitoren bewirken eine Akkumulation des intrazellulären cAMP-Spiegels, wodurch die Produktion von entzündungsfördernden Zytokinen unterdrückt und die Produktion von entzündungshemmenden Mediatoren erhöht wird.^11^ Im Gegensatz zu anderen Therapeutika, die direkt auf Zytokine abzielen, greifen PDE4-Inhibitoren an einem früheren Punkt der Entzündungskaskade ein.

Apremilast ist für die Behandlung der Plaque-Psoriasis, der psoriatischer Arthritis und oralen Geschwüren bei Morbus Behçet zugelassen. Außerdem gibt es Hinweise darauf, dass Apremilast auch bei der Behandlung der AD wirksam ist. Die klinischen Studien wurden jedoch abgebrochen, da bei Patienten, die zweimal täglich 40 mg Apremilast erhielten, eine höhere Zellulitis-Inzidenz auftrat.^12^ Bemerkenswert ist, dass Immunzellen bei AD hohe Mengen an PDE-4 exprimieren und dass AD-Mausmodelle gut auf die Behandlung mit Apremilast ansprechen.^13–15^ Da das nummuläre Ekzem sowohl mit der Psoriasis als auch mit der AD klinische Überschneidungen aufweist, wurde die Hypothese aufgestellt, dass Apremilast die klinischen Manifestationen des NE wirksam reduzieren könnte.

**PATIENTEN UND METHODIK**

**Aufbau der Studie**

Zur Untersuchung der Wirksamkeit und Sicherheit von Apremilast beim nummulären Ekzem (NE) führten wir eine prospektive, randomisierte, doppelblinde, placebokontrollierte, monozentrische Phase-IIb-Studie durch (Abbildung 1). Ursprünglich war geplant, bis zu 40 Patienten nach schriftlicher Einverständniserklärung in die Studie aufzunehmen. Die Patienten erhielten in der verblindeten Phase zwischen Woche 0 und 16 entweder Apremilast 30 mg zweimal täglich oder ein entsprechendes Placebo. Danach wurden alle Patienten bis Woche 32 mit Apremilast 30 mg zweimal täglich behandelt. Während der Screening- und Behandlungsphase wurde die durchschnittliche Menge an topischen Kortikosteroiden (TCS) (Klasse 2) ermittelt. Die Studie wurde von der zuständigen Ethikkommission und dem Bundesinstitut für Arzneimittel und Medizinprodukte genehmigt und vor Studienbeginn auf unter *ClinicalTrials.gov* registriert (NCT03160248).

**Primärer Endpunkt**

Der primäre Endpunkt war die Anzahl der Patienten, die entweder eine Verbesserung des *Physician’s Global Assessment* (PGA) um mindestens zwei Punkte auf der 5-Punkte-Skala zwischen Woche 0 (Baseline) und Woche 16 erreichten oder in Woche 16 einen absoluten PGA-Wert von 0 oder 1 aufwiesen.

**Sekundäre Endpunkte**

Die sekundären Endpunkte wurden in Woche 16 und 32 bewertet. Dazu zählten eine Reduktion des *Eczema Area and Severity Index* (EASI) um 50 %, Veränderungen des transepidermalen Wasserverlusts (TEWL), gemessen mit einem Tewameter^®^ TM Hex, eine Abnahme der Akanthose oder der Anzahl inflammatorischer Zellen in Hautbiopsien, beurteilt durch einen zertifizierten dermatopathologischen Gutachter, Veränderungen der verwendeten Menge topischer Kortikosteroide (TCS), Veränderungen im *Physician’s Global Assessment* (PGA), im *Dermatology Life Quality Index* (DLQI), in der visuellen Analogskala (VAS) für Pruritus, in der Subskala zur globalen Zufriedenheit des *Treatment Satisfaction Questionnaire for Medication* (TSQM) sowie Angaben zur Sicherheit.

**Einschluss- und Ausschlusskriterien**

Wichtige Einschlusskriterien waren eine klinisch und histologisch bestätigte moderate bis schwere Neurodermitis (PGA 3–5), die trotz Anwendung topischer Kortikosteroide (TCS) über mindestens acht Wochen unzureichend kontrolliert war. Die Erkrankung musste seit mindestens drei Monaten bestehen. Die histologische Diagnose eines Ekzems erfolgte nach Ermessen des dermatopathologischen Gutachters und umfasste typische Merkmale wie Spongiose, Eosinophile, Serumkrusten und (unregelmäßige) Akanthose sowie das Fehlen charakteristischer Merkmale einer Differenzialdiagnose (zum Beispiel Munro-Mikroabszesse). Zentrale Ausschlusskriterien waren begleitende schwere Erkrankungen, darunter Malignome, immunologische oder neurologische Störungen, Depressionen oder Suizidgedanken, kardiovaskuläre Erkrankungen sowie schwere Nieren- oder Leberinsuffizienz.

**Berechnung des Stichprobenumfangs**

Unter der Annahme einer Verbesserung um 2 Punkte (Standardabweichung 1,7) in der Apremilast-Gruppe im Vergleich zu 0 Punkten in der Placebo-Gruppe wurde eine Stichprobengröße von 17 Patienten pro Studienarm geschätzt, um mit Hilfe des zweiseitigen Wilcoxon-Tests mit einer Signifikanz von 5 % einen Unterschied in den medianen PGA-Scores mit einer Power von 85 % nachzuweisen. Die Abbrecherquote wurde auf 15 % geschätzt, so dass eine Rekrutierung von 40 Patienten (20 pro Gruppe) geplant war*.*

**Plan für die statistische Analyse**

Alle Patienten, die mindestens eine Dosis der Studienmedikation erhielten, wurden in die endgültige Analyse einbezogen. Für den primären Endpunkt wurde die absolute Veränderung des PGA-Scores in Woche 16 zwischen der Apremilast- und der Placebogruppe anhand des zweiseitigen Wilcoxon-Tests mit einem Signifikanzniveau von 5 % verglichen. Fehlende Daten wurden unter Verwendung der höchsten dokumentierten Veränderung des PGA-Wertes in der Placebogruppe und 0 in der Apremilast-Gruppe korrigiert.

**ERGEBNISSE**

**Demografische und Ausgangsdaten der Patienten**

Gemäß dem Studienprotokoll sollten 40 Patienten in die Studie aufgenommen werden. Da sich die Rekrutierung verzögerte, beschlossen die Studienleiter, die weitere Rekrutierung nach der Aufnahme von 33 Patienten zu beenden. Zwei Patienten zogen ihre Einwilligung vor der Randomisierung zurück. Einunddreißig Patienten begannen die Behandlung (15 in der Apremilast-Gruppe, 15 in der Placebogruppe). Neunzehn Patienten nahmen bis zum Ende der Studie teil, während 12 Patienten die Studie aufgrund von unerwünschten Ereignissen (n = 5) oder Rücknahme der Einwilligung (n = 7) vorzeitig abbrachen (5 im Apremilast-Arm, 7 im Placebo-Arm). In der Apremilast-Gruppe schlossen elf von 15 Patienten (73,3 %) die verblindete Phase ab, in der zunächst mit Placebo behandelten und anschließend auf Apremilast umgestellten Vergleichsgruppe waren es 15 von 16 Patienten (93,8 %). Bis zum Ende der Open-Label-Phase verblieben zehn von 15 Patienten (66,7 %) beziehungsweise neun von 16 Patienten (56,3 %) im Studienverlauf. Zwischen der Apremilast- und der initial mit Placebo behandelten Vergleichsgruppe bestanden keine signifikanten Unterschiede hinsichtlich der mittleren PGA-Werte oder der Ausgangsdaten (Tabelle 1).

**Apremilast ist keine wirksame Behandlung für nummuläres Ekzem**

Es gab keinen statistischen Unterschied zwischen der Apremilast-Gruppe und der Placebo-Gruppe in Bezug auf den primären Endpunkt (Verbesserung des PGA um 2 Punkte bis Woche 16). Aufgrund fehlender Werte wurde der PGA-Wert in Woche 16 für vier Patienten berechnet, wie im Abschnitt Methoden beschrieben. Eine Verbesserung des PGA wurde bei 1/15 (6,7 %) Patienten in der Apremilast-Gruppe und 4/16 (25,0 %) Patienten in der Placebo-Gruppe beobachtet (p = 0,369) (Tabelle 2). Bei der Analyse ausschließlich der abgeschlossenen Fälle ohne Imputation zeigte sich eine PGA-Verbesserung bei einem von elf Patienten (9,1 %) in der Apremilast-Gruppe und bei zwei von 14 Patienten (14,3 %) in der zunächst mit Placebo behandelten und anschließend auf Apremilast umgestellten Vergleichsgruppe (p = 0,812) (Tabelle S1, Online-Supplement).

Somit zeigte sich kein Unterschied in der Verbesserung des PGA zwischen den mit Apremilast behandelten Patienten und jenen, die Placebo erhielten. Diese Studie umfasste eine Reihe sekundärer Endpunkte, wie EASI50 (Tabelle 1), die Verbesserung der Hautentzündung in Hautbiopsien in Woche 0 und 16, Veränderungen im täglichen Gebrauch topischer Kortikosteroide, Lebensqualität, Juckreiz und transepidermaler Wasserverlust sowie die Gesamtzufriedenheit mit der Behandlung mit Apremilast, gemessen mit dem Fragebogen zur Behandlungszufriedenheit mit Medikamenten (Treatment Satisfaction Questionnaire for Medication; TSQM). Keiner dieser sekundären Endpunkte zeigte einen Unterschied zwischen Apremilast und Placebo in Woche 16 oder Woche 32 (Tabellen S2–S8, Online-Supplement). Zusammenfassend lässt sich sagen, dass es keine Belege für eine Wirksamkeit von Apremilast bei NE gibt.

**Apremilast wurde gut vertragen und es traten keine neuen Sicherheitssignale auf**

Insgesamt wurden 59 unerwünschte Ereignisse (adverse events, AE) gemeldet. Bei 37,3 % der AEs wurde ein Zusammenhang mit der verabreichten Behandlung angenommen. Während der verblindeten Phase traten in der Apremilast-Gruppe 22 und in der Placebo-Gruppe 21 unerwünschte Ereignisse auf. Während der offenen Phase wurden zusätzlich 16 weitere AE gemeldet. In der Apremilast-Gruppe kam es während der verblindeten Phase zu einem schwerwiegenden unerwünschten Ereignis (serious AE, SAE), bei dem ein Patient aufgrund einer Verschlechterung der Neurodermitis hospitalisiert werden musste (Tabelle 3). Die Mehrheit der AE wurde als leicht (61 %) oder moderat (35,6 %) eingestuft; lediglich zwei Ereignisse (3,4 %) galten als schwerwiegend. Es traten keine unerwarteten schwerwiegenden unerwünschten Ereignisse, keine lebensbedrohlichen und keine tödlichen Ereignisse auf. Unter Apremilast kam es häufiger zu Diarrhö, Übelkeit und viralen Infektionen der oberen Atemwege als unter Placebo. Eine vollständige Auflistung aller AE ist in den Tabellen S9 und S10 im Online-Supplement zu finden. Das Gesamtsicherheitsprofil entsprach dem bislang bekannten Sicherheitsprofil von Apremilast.

**DISKUSSION**

Entzündliche Hauterkrankungen schränken die Lebensqualität der Patienten erheblich ein.^16,17^ Zwar wurden zahlreiche neue Wirkstoffe zur Behandlung der beiden häufigsten entzündlichen Hauterkrankungen, Psoriasis und atopische Dermatitis, zugelassen, doch die große Mehrheit der dieser Erkrankungen bleibt eine therapeutische Herausforderung.^18^ Das nummuläre Ekzem ist ein herausragendes Beispiel für eine entzündliche Hauterkrankungen, die trotz ihrer hohen Prävalenz bei Kindern und Erwachsenen bisher nur unzureichend untersucht wurde. Zweifellos handelt es sich bei NE um eine relevante entzündliche Hauterkrankung mit einem hohen ungedeckten Bedarf an neuen therapeutischen Optionen. Die Erforschung des nummulären Ekzems wird jedoch durch uneinheitliche Definitionen, die Verwendung mehrerer Synonyme sowie die begrenzte Datenlage in der Literatur erheblich erschwert.

Hier berichten wir über die erste randomisierte kontrollierte klinische Studie bei Patienten mit NE. Die Patienten erhielten 16 Wochen lang 30 mg Apremilast zweimal täglich oder Placebo in einer verblindeten Phase, gefolgt von einer offenen Phase über weitere 16 Wochen. Da für das NE kein spezifischer klinischer Score existiert, wurde eine signifikante Verbesserung im 5-Punkte-PGA als primärer Endpunkt definiert. Ergänzend wurden mehrere sekundäre Endpunkte berücksichtigt, um potenzielle zusätzliche oder subtilere Unterschiede zwischen der Behandlung mit Placebo und Apremilast zu verschiedenen Zeitpunkten zu erfassen. Apremilast zeigte keine Wirksamkeit beim nummulären Ekzem – weder in Bezug auf die Verbesserung des PGA noch hinsichtlich krankheitsbezogener Symptome, der Lebensqualität oder histologischer Veränderungen.

Unsere Studie hat Einschränkungen. Erstens wurde das Rekrutierungsziel von 40 Patienten nicht erreicht. In Anbetracht der eindeutigen Ergebnisse ist jedoch nicht zu erwarten, dass sich die Ergebnisse bei einer höheren Patientenzahl wesentlich ändern würden. Zweitens gab es eine hohe Abbrecherquote, die höchstwahrscheinlich auf die geringe Wirksamkeit zurückzuführen ist. Und schließlich handelt es sich um eine Studie an einem einzigen Zentrum in Deutschland, die nicht das Spektrum der verschiedenen Ethnien repräsentiert. Dennoch sind die Ergebnisse dieser Studie von großer Bedeutung, da sie einen wichtigen Beitrag zur laufenden Debatte darüber leisten, ob es sich bei der NE um eine Variante der AD mit einer vorherrschenden Th2-Immunantwort, einen gemischten Th2/Th17-Phänotyp oder eine eigenständige Entität mit einer noch zu entdeckenden Immunpathogenese handelt.

Das nummuläre Ekzem weist klinische, histologische und molekulare Merkmale sowohl der Typ-2- (Th2) als auch der Typ-3-Immunität (Th17) auf.^19^ Klinisch weist das nummuläre Ekzem eine scharfe Begrenzung und ähnliche Lokalisationen wie die Psoriasis auf, wobei einzelne Läsionen häufig Papeln und Serumkrusten zeigen. Die histologischen Veränderungen des NE bestehen aus Merkmalen sowohl des Ekzems (Spongiose, Eosinophile) als auch der Psoriasis (Akanthose, Neutrophile). Juckreiz und mikrobielle Besiedlung deuten auf eine Ekzemvariante hin.^4,5,20–22^ Aus immunologischer Sicht sind in der läsionalen Haut des nummulären Ekzems sowohl Typ-2-assoziierte Gene wie *IL13*, *CCL17*, *CCL18* und *CCL26* als auch Typ-3-assoziierte Gene wie *IL19*, *CXCL8* und *CXCL5* hochreguliert.^19^ Es bleibt jedoch unklar, ob die Th2- und Th17-Immunität ko-dominant vorliegen oder ob eine der beiden Immunsignaturen dem nummulären Ekzem ursächlich zugrunde liegt. Hier können ausschließlich Daten aus randomisierten, kontrollierten klinischen Studien endgültig Aufschluss darüber geben, ob und in welchem Ausmaß die unterschiedlichen Immunmechanismen für das nummuläre Ekzem relevant sind.

In unserer Studie untersuchten wir die Wirksamkeit und Sicherheit von Apremilast, einem kleinen Molekül, das PDE4 hemmt und dadurch die Th17-Immunität sowie die NF-kB-getriebene Entzündung dämpft. Angesichts der mangelnden Wirksamkeit bei NE und der Tatsache, dass Apremilast für die Behandlung von Plaque-Psoriasis, einem Prototyp Th17-vermittelter Hauterkrankungen, zugelassen ist, können wir daraus schließen, dass die Th17-Immunität bei der Immunpathogenese von NE nicht die dominierende Rolle spielt. Diese Beobachtung steht im Einklang mit klinischen Studien, in denen Apremilast bei AD getestet wurde. Hier zeigte nur die höchste Dosierung von 40 mg Apremilast BID klinische Verbesserungen. Dieser Arm musste jedoch aufgrund von Sicherheitsbedenken eingestellt warden.^12^ Auf der Grundlage unserer Daten sind neue therapeutische Optionen für NE denkbar. Die Hemmung der Th2-Zytokine durch Dupilumab, einen IL-4/IL-13-Rezeptor-Antagonisten, sowie durch die gegen IL-13 gerichteten Antikörper Tralokinumab beziehungsweise Lebrikizumab erscheint vielversprechend. Auch der Einsatz von Janus-Kinase-Inhibitoren, die für die Behandlung von AD zugelassen sind (Baricitinib, Upadacitinib, Abrocitinib), könnte von Vorteil sein. Es sei darauf hingewiesen, dass derzeit eine von Prüfärzten initiierte klinische Studie läuft, in der die Wirksamkeit von Dupilumab beim NE untersucht wird (clinicaltrials.gov-Kennung: NCT04600362). Zusammenfassend haben wir gezeigt, dass Apremilast für die Behandlung des NE nicht wirksam ist, was beweist, dass eine mögliche Aktivierung der Th17-Immunität für die Entstehung oder Aufrechterhaltung des NE nicht ursächlich ist. Andere Therapieschemata, die die Th2-Immunität hemmen, scheinen vielversprechender zu sein, und künftige Studien sind erforderlich, um bessere Behandlungsstrategien für das NE zu definieren.

**DANKSAGUNG**

Wir danken allen Patienten für ihre Studienteilnahme sowie dem Münchener Studienzentrum für die hervorragende Unterstützung bei Monitoring, Datenmanagement und statistischer Auswertung.

**INTERESSENKONFLIKT**

F.L. erhielt Honorare für Vorträge und/oder Tätigkeiten als Mitglied von Advisory Boards von AbbVie, Novartis, LEO Pharma, Lilly, Roche, Sanofi, Almirall, Janssen, Amgen, UCB, Boehringer Ingelheim, Bristol Myers Squibb und Union Therapeutics. K.E. erhielt Honorare für Vorträge und/oder Tätigkeiten als Mitglied von Advisory Boards von AbbVie, Almirall, Bristol Myers Squibb, Boehringer Ingelheim, LEO Pharma, Lilly, Janssen, Novartis, Pfizer, Sanofi und UCB.

Literatur

1. Bonamonte D, Foti C, Vestita M, et al. Nummular eczema and contact allergy: a retrospective study. *Dermatitis.* 2012;23:153-157.
2. Halberg M. Nummular eczema. *J Emerg Med*. 2012;43:e327-e328.
3. Devergie M-G-A. Traité pratique des maladies de la peau, 1854.
4. Jiamton S, Tangjaturonrusamee C, Kulthanan K. Clinical features and aggravating factors in nummular eczema in Thais. *Asian Pac J Allergy Immunol.* 2013;31:36-42.
5. Kim WJ, Ko HC, Kim MB, et al. Features of *Staphylococcus aureus* colonization in patients with nummular eczema. *Br J Dermatol.* 2013;168:658-660.
6. Silverberg JI, Hou A, Warshaw EM, et al. Prevalence and trend of allergen sensitization in patients with nummular (discoid) eczema referred for patch testing: North American Contact Dermatitis Group data, 2001–2016. *Contact Dermatitis.* 2021;85:46-57.
7. Shen Y, Pielop J, Hsu S. Generalized nummular eczema secondary to peginterferon Alfa-2b and ribavirin combination therapy for hepatitis C infection. *Arch Dermatol.* 2005;141: 102-103.
8. Noda S, Suarez-Farinas M, Ungar B, et al. The Asian atopic dermatitis phenotype combines features of atopic dermatitis and psoriasis with increased TH17 polarization. *J Allergy Clin Immunol.* 2015;136:1254-1264.
9. Roberts H, Orchard D. Methotrexate is a safe and effective treatment for paediatric discoid (nummular) eczema: a case series of 25 children. *Australas J Dermatol*. 2010;51:128-130.
10. Moustafa F, Feldman SR. A review of phosphodiesterase-inhibition and the potential role for phosphodiesterase 4-inhibitors in clinical dermatology. *Dermatol Online J*. 2014;20: 22608.
11. Schett G. Apremilast in psoriatic arthritis. *Clin Exp Rheumatol.* 2015;33:S98-S100.
12. Simpson EL, Imafuku S, Poulin Y, et al. A Phase 2 Randomized Trial of Apremilast in Patients with Atopic Dermatitis*. J Invest Dermatol.* 2019;139:1063-1072.
13. Chan SC, Reifsnyder D, Beavo JA, Hanifin JM. Immunochemical characterization of the distinct monocyte cyclic AMP-phosphodiesterase from patients with atopic dermatitis. *J allergy Clin Immunol*. 1993;91:1179-1188.
14. Baumer W, Hoppmann J, Rundfeldt C, Kietzmann M. Highly selective phosphodiesterase 4 inhibitors for the treatment of allergic skin diseases and psoriasis. *Inflamm Allergy Drug Targets.* 2007;6:17-26.
15. Schafer PH, Adams M, Horan G, et al. Apremilast Normalizes Gene Expression of Inflammatory Mediators in Human Keratinocytes and Reduces Antigen-Induced Atopic Dermatitis in Mice. *Drugs R D.* 2019;19:329-338.
16. Misery L, Seneschal J, Reguiai Z, et al. Patient Burden is Associated with Alterations in Quality of Life in Adult Patients with Atopic Dermatitis: Results from the ECLA Study. *Acta Derm Venereol.* 2018;98(7):713-714.
17. Langenbruch A, Radtke MA, Jacobi A, et al. Quality of psoriasis care in Germany: results of the national health care study “PsoHealth3”. *Arch Dermatol Res.* 2016;308:401-408.
18. Eyerich K, Eyerich S. Immune response patterns in non-communicable inflammatory skin diseases. *J Eur Acad Dermatol Venereol*. 2018;32:692-703.
19. Bohner A, Jargosch M, Muller NS, et al. The neglected twin: Nummular eczema is a variant of atopic dermatitis with codominant T(H)2/T(H)17 immune response. *J Allergy Clin Immunol.* 2023;152:408-419
20. Rendon A, Schakel K. Psoriasis Pathogenesis and Treatment. *Int J Mol Sci.* 2019;20.
21. Kong HH, Oh J, Deming C, Conlan S, et al. Temporal shifts in the skin microbiome associated with disease flares and treatment in children with atopic dermatitis. *Genome Res*. 2012;22:850-859.
22. Weidinger S, Novak N. Atopic dermatitis. *Lancet.* 2016;387:1109-1122.

TABELLE 1 Demografische und Ausgangsmerkmale.

TABELLE 2 Primärer Endpunkt: *Physician’s Global Assessment* (PGA) sowie 50%ige Verbesserung des *Eczema Area and Severity Index* (EASI50) in Woche 16 und 32.

TABELLE 3 Unerwünschte Ereignisse (AE) und schwerwiegende unerwünschte Ereignisse (SAE).

[[To Aptara: Lettering in figures should always be in lower-case type (a, b, c etc.).]]

ABBILDUNG 1 (a) *Consort Flow Diagram* der eingeschlossenen Patienten; (b) Studiendesign.

[[Online-Supplement]]

TABELLE S1 Verbesserung der *Physician’s Global Assessment* (PGA), nur vollständige Fälle bis Woche 16.

TABELLE S2 Veränderung des mittleren transepidermalen Wasserverlusts (TEWL).

TABELLE S3 Histologische Verbesserung.

ABELLE S4 Mittlerer täglicher Verbrauch topischer Steroide (g).

TABELLE S5 *Dermatology Life Quality Index* (DLQI).

TABELLE S6 Pruritus visuelle Analogskala (VAS) in Woche 16 und 32.

TABELLE S7 *Treatment Satisfaction Questionnaire for Medication* (TSQM)-Score.

TABELLE S8 Veränderung der *Physician’s Global Assessment* (PGA) zwischen verblindeter und offener Phase.

TABELLE S9 Nicht schwerwiegende unerwünschte Ereignisse – verblindete Phase.

TABELLE S10 Nicht schwerwiegende unerwünschte Ereignisse – offene Phase.

Tabelle 1

|  | | **Behandlungsgruppe** | | | |
| --- | --- | --- | --- | --- | --- |
|  |  | ***Apremilast***  ***(n = 15)*** | | ***Placebo (w16–32 Apremilast)***  ***(n = 16)*** | |
| Geschlecht (n, %) | Weiblich | 5 | (33,3 %) | 2 | (12,5 %) |
|  | Männlich | 10 | (66,7 %) | 14 | (87,5 %) |
| Altersgruppe (n, %) | Erwachsene (18–64) | 11 | (73,3 %) | 11 | (68,8 %) |
|  | Ältere Patienten (65–84) | 4 | (26,7 %) | 5 | (31,5 %) |
| Alter (Jahre) | Mittelwert | 54,5 |  | 54,6 |  |
|  | Standardabweichung | 17,0 |  | 15,5 |  |
|  | Minimum | 27,0 |  | 23,0 |  |
|  | Median | 62,0 |  | 55,5 |  |
|  | Maximum | 81,0 |  | 78,0 |  |
| Ethnie (n, %) | Asiatisch | 1 | (6,7 %) | 1 | (6,3 %) |
|  | Weiß | 13 | (86,7 %) | 15 | (93,8 %) |
|  | Andere | 1 | (6,7 %) | 0 |  |
| BMI | Mittelwert | 28,7 |  | 28,6 |  |
|  | Standardabweichung | 4,8 |  | 6,0 |  |
| PGA | 3 | 12 | (80,0 %) | 15 | (20,0 %) |
|  | 4 | 3 | (20,0 %) | 1 | (6,3 %) |

Tabelle 2

| **PGA-Verbesserung zum Ausgangsbefund** | |  | | | |  |
| --- | --- | --- | --- | --- | --- | --- |
|  |  | ***Apremilast*** | | ***Placebo + Apremilast*** | | ***p-Wert*** |
|  |  | (n = 15) | | (n = 16) | |  |
| Woche 16 | Keine Verbesserung | 14 | (93,3 %) | 12 | (75,0 %) | 0,369 |
|  | Verbesserung | 1 | (6,7 %) | 4 | (25,0 %) |  |
| **EASI 50 Verbesserung zum Ausgangswert** | |  | | | |  |
|  |  | ***Apremilast*** | | ***Placebo + Apremilast*** | | ***p-Wert*** |
|  |  | (n = 11) | | (n = 14) | |  |
| Woche 16 | Keine Verbesserung | 6 | (54,5 %) | 8 | (57,1 %) | 1,000 |
|  | Verbesserung | 5 | (45,5 %) | 6 | (42,9 %) |  |
|  |  | (n = 11) | | (n = 11) | |  |
| Woche 32 | Keine Verbesserung | 6 | (54,5 %) | 5 | (45,5 %) | 0,670 |
|  | Verbesserung | 5 | (45,5 %) | 6 | (54,5 %) |  |

Tabelle 3.

|  | **Verblindete Phase**  **________________________________** | | | | | | **Offene Phase**  **_______________** | | |
| --- | --- | --- | --- | --- | --- | --- | --- | --- | --- |
|  | ***Apremilast n = 15 _______________*** | | | ***Placebo n = 16 _______________*** | | | ***Apremilast n = 25 _______________*** | | |
|  | *Ereignisse* | *n* | *( %)* | *Ereignisse* | *n* | *( %)* | *Ereignisse* | *n* | *( %)* |
| Alle AEs | 22 | 10 | (67) | 21 | 10 | (63) | 16 | 9 | (36) |
| SAE | 1 | 1 | (7) | 0 | 0 |  | 0 | 0 |  |
| Non-SAE AEs | 21 | 10 | (67) | 21 | 10 | (63) | 16 | 9 | (36) |

**[[Supplementary tables]]**

Tabelle S1

| **PGA-Verbesserung zum Ausgangsbefund** | | **Behandlungsgruppe** | | | |  |
| --- | --- | --- | --- | --- | --- | --- |
|  |  | **Apremilast** | | **Placebo + Apremilast** | | **p-Wert** |
|  |  | (n = 11) | | (n = 14) | |  |
| Woche 16  (Abgeschlossene Fälle) | Keine Verbesserung | 10 | (90,9 %) | 12 | (85,7 %) | 0,812 |
|  | Verbesserung | 1 | (9,1 %) | 2 | (14,3 %) |  |

Tabelle S2

| **Veränderung mittlerer TEWL zwischen Ausgangsbefund und …** | |  | | **p-Wert** |
| --- | --- | --- | --- | --- |
|  |  | **Apremilast** | **Placebo + Apremilast** |  |
| Woche 16 | n | 9 | 9 | 0,185 |
|  | Mittelwert | –26,04 | –12,24 |  |
|  | Std | 10,29 | 21,47 |  |
| Woche 32 | n | 8 | 6 | 0,847 |
|  | Mittelwert | –19,08 | –16,85 |  |
|  | Std | 19,46 | 29,73 |  |

Tabelle S3

| **Histologische Verbesserung** | |  | | **p-Wert**  **(Fisher Exact Test)** |
| --- | --- | --- | --- | --- |
|  |  | **Apremilast** | **Placebo + Apremilast** |  |
| Gesamtverbesserung  at Week 16 | Keine Verbesserung | 7 (63,6 %) | 9 (64,3 %) | 1,000 |
|  | Verbesserung | 4 (36,4 %) | 5 (35,7 %) |  |
| Epidermale Dicke (Ausgangsbefund) | n | 11 | 14 |  |
|  | Mittelwert | 318,73 | 291,29 |  |
|  | Std | 88,84 | 114,44 |  |
| Epidermale Dicke  (Woche 16) | n | 11 | 14 |  |
|  | Mittelwert | 230,32 | 200,57 |  |
|  | Std | 59,29 | 127,07 |  |
| Immunzell-Zahl (Ausgangsbefund) | n | 11 | 14 |  |
|  | Mittelwert | 192,77 | 193,11 |  |
|  | Std | 59,08 | 69,08 |  |
| Immunzell-Zahl  (Week 16) | n | 11 | 14 |  |
|  | Mittelwert | 115,55 | 112,00 |  |
|  | Std | 82,74 | 89,38 |  |

Tabelle S4

| **Mittlerer täglicher Steroidverbrauch während …** | |  | | **p-Wert** |
| --- | --- | --- | --- | --- |
|  |  | **Apremilast** | **Placebo + Apremilast** |  |
| Screening | n | 13 | 13 | 0,538 |
|  | Mittelwert | 4,23 | 2,63 |  |
|  | Std | 3,79 | 1,44 |  |
| Phase I (bis Woche 16) | n | 13 | 15 | 0,854 |
|  | Mittelwert | 3,18 | 2,84 |  |
|  | Std | 3,02 | 1,85 |  |
| Phase II (Woche 16–Woche 32) | n | 11 | 10 | 0,597 |
|  | Mittelwert | 1,31 | 1,61 |  |
|  | Std | 1,10 | 1,10 |  |

Tabelle S5

| **Veränderung des DLQI zwischen Ausgangsbefund und …** | |  | | **p-Wert** |
| --- | --- | --- | --- | --- |
|  |  | **Apremilast** | **Placebo + Apremilast** |  |
| Woche 16 | n | 11 | 14 | 0,678 |
|  | Mittelwert | –3,82 | –2,36 |  |
|  | Std | 4,92 | 3,39 |  |
| Woche 32 | n | 11 | 11 | 0,792 |
|  | Mittelwert | –4,45 | –4,64 |  |
|  | Std | 9,46 | 5,71 |  |

Tabelle S6

| **Veränderung der VAS zwischen Ausgangsbefund und …** | |  | | **p-Wert** |
| --- | --- | --- | --- | --- |
|  |  | **Apremilast** | **Placebo + Apremilast** |  |
| Woche 16 | n | 11 | 14 | 0,617 |
|  | Mittelwert | –0,27 | –0,18 |  |
|  | Std | 2,38 | 1,69 |  |
| Woche 32 | n | 11 | 11 | 0,741 |
|  | Mittelwert | –1,00 | –1,55 |  |
|  | Std | 4,04 | 2,94 |  |

Tabelle S7

| **Veränderung des TSQM zwischen Ausgangsbefund und …** | |  | | **p-Wert** |
| --- | --- | --- | --- | --- |
|  |  | **Apremilast** | **Placebo + Apremilast** |  |
| Wirksamkeit  Woche 16 | n | 9 | 10 | 0,152 |
|  | Mittelwert | –3,71 | –12,24 |  |
|  | Std | 20,22 | 30,75 |  |
| Wirksamkeit  Woche 32 | n | 9 | 8 | 0,194 |
|  | Mittelwert | –0,01 | 19,84 |  |
|  | Std | 32,62 | 24,44 |  |
| Nebenwirkungen  Woche 16 | n | 9 | 10 | 0,428 |
|  | Mittelwert | –6,94 | 2,50 |  |
|  | Std | 52,70 | 29,93 |  |
| Nebenwirkungen  Woche 32 | n | 9 | 8 | 0,285 |
|  | Mittelwert | –13,88 | –34,11 |  |
|  | Std | 52,73 | 37,75 |  |
| Anwendungskomfort  Woche 16 | n | 9 | 10 | 0,775 |
|  | Mittelwert | 15,42 | 10,04 |  |
|  | Std | 32,11 | 29,32 |  |
| Anwendungskomfort  Woche 32 | n | 9 | 8 | 0,661 |
|  | Mittelwert | 9,87 | 14,59 |  |
|  | Std | 48,00 | 26,76 |  |
| Gesamt-Zufriedenheit Woche 16 | n | 9 | 10 | 0,367 |
|  | Mittelwert | –10,31 | –21,36 |  |
|  | Std | 25,77 | 42,59 |  |
| Gesamt-Zufriedenheit Week 32 | n | 9 | 8 | 0,665 |
|  | Mittelwert | –2,38 | 2,72 |  |
|  | Std | 37,61 | 36,63 |  |

Tabelle S8

| **Veränderung des PGA zwischen …** | |  | |
| --- | --- | --- | --- |
|  |  | **Apremilast** | **Placebo + Apremilast** |
| p-Wert (Wilcoxon Signed Ranks Test)  Vergleich zwischen verblindeter und offener Phase | | 0,391 | 0,625 |
| Ausgangsbefund und Woche 16 | n | 11 | 14 |
|  | Mittelwert | –0,73 | –0,57 |
|  | Std | 0,90 | 0,85 |
| Ausgangsbefund und Woche 32 | n | 11 | 11 |
|  | Mittelwert | –0,73 | –0,82 |
|  | Std | 0,65 | 0,87 |
| Woche 16 und Woche 32 | n | 11 | 11 |
|  | Mittelwert | 0,00 | –0,55 |
|  | Std | 1,18 | 0,82 |

Tabelle S9

|  | **Apremilast exponiert n = 15 _______________** | | | **Placebo exponiert n = 16 _______________** | | |
| --- | --- | --- | --- | --- | --- | --- |
| **System Organ Klasse** |  |  |  |  |  |  |
|  | **Ereignisse** | **n** | **( %)** | **Ereignisse** | **n** | **( %)** |
| ***Gesamt*** | 21 | 10 | (67) | 21 | 10 | (63) |
| ***Gastrointestinale Erkrankungen*** | 9 | 7 | (47) | 2 | 2 | (13) |
| Blähungen | 1 | 1 | (7) | 0 | 0 | (0) |
| Abdominelle Schmerzen | 1 | 1 | (7) | 0 | 0 | (0) |
| Diarrhoe | 3 | 3 | (20) | 1 | 1 | (6) |
| Flatulenz | 1 | 1 | (7) | 0 | 0 | (0) |
| Nausea | 3 | 3 | (20) | 1 | 1 | (6) |
| ***Generelle Veränderungen und lokale Reaktionen*** | 2 | 1 | (7) | 1 | 1 | (6) |
| Schmerzen im Brustkorb | 0 | 0 | (0) | 1 | 1 | (6) |
| Fatigue | 2 | 1 | (7) | 0 | 0 | (0) |
| ***Infektionen*** | 7 | 7 | (47) | 4 | 3 | (19) |
| Hordeolum | 1 | 1 | (7) | 0 | 0 | (0) |
| Pneumonie | 0 | 0 | (0) | 1 | 1 | (6) |
| Virale Infektion der oberen Atemwege | 6 | 6 | (40) | 3 | 3 | (19) |
| ***Laborwerte*** | 1 | 1 | (7) | 0 | 0 | (0) |
| Erhöhung der Leberwerte | 1 | 1 | (7) | 0 | 0 | (0) |
| ***Metabolismus und Ernährung*** | 1 | 1 | (7) | 0 | 0 | (0) |
| Reduzierter Appetit | 1 | 1 | (7) | 0 | 0 | (0) |
| ***Muskuloskelettal Erkrankungen und Bindegewebserkrankungen*** | 0 | 0 | (0) | 1 | 1 | (6) |
| Muskelschmerz | 0 | 0 | (0) | 1 | 1 | (6) |
| ***Benigne Neoplasien (inklusive Zysten und Polypen)*** | 0 | 0 | (0) | 1 | 1 | (6) |
| Keratoakanthom | 0 | 0 | (0) | 1 | 1 | (6) |
| ***Nervensystem-Erkrankungen*** | 1 | 1 | (7) | 3 | 2 | (13) |
| Benommenheit | 1 | 1 | (7) | 0 | 0 | (0) |
| Kopfschmerz | 0 | 0 | (0) | 1 | 1 | (6) |
| Migraine | 0 | 0 | (0) | 2 | 1 | (6) |
| ***Hauterkrankungen*** | 0 | 0 | (0) | 9 | 3 | (19) |
| Ekzem | 0 | 0 | (0) | 1 | 1 | (6) |
| Ekzem Verschlechterung | 0 | 0 | (0) | 4 | 1 | (6) |
| Pruritus | 0 | 0 | (0) | 4 | 2 | (13) |

Tabelle S10

|  | **Apremilast exponiert n = 25 _______________** | | |
| --- | --- | --- | --- |
| **System Organ Klasse** |  |  | |
|  | **Events** | **n** | **( %)** |
| ***Gesamt*** | 16 | 9 | (36) |
| ***Augenerkrankungen*** | 1 | 1 | (4) |
| Katarakt | 1 | 1 | (4) |
| ***Gastrointestinale Erkrankungen*** | 6 | 4 | (16) |
| Blähungen | 1 | 1 | (4) |
| Diarrhoe | 3 | 2 | (8) |
| Nausea | 1 | 1 | (4) |
| Erbrechen | 1 | 1 | (4) |
| ***Generelle Veränderungen und lokale Reaktionen*** | 1 | 1 | (4) |
| Periphere Ödeme | 1 | 1 | (4) |
| ***Infektionen*** | 2 | 2 | (8) |
| Virale Infektion der oberen Atemwege | 2 | 2 | (8) |
| ***Erkrankungen des Nervensystems*** | 2 | 2 | (8) |
| Kopfschmerz | 2 | 2 | (8) |
| ***Hautveränderungen*** | 4 | 2 | (8) |
| Ekzem | 1 | 1 | (4) |
| Photodermatose | 1 | 1 | (4) |
| Pruritus | 2 | 2 | (8) |
